# Genome-wide analysis of the *GATA* gene family in *Castanea mollissima* reveals key regulators involved in salt stress response

**DOI:** 10.3389/fpls.2026.1907055

**Published:** 2026-10-12

**Authors:** Xiangyu Wang, Xili Liu, Jun Liu, Le Niu, Huoqiang Quan, Tong Zhang, Qinuo Li, Guoyun Zhang, Liyang Yu

**Affiliations:** 1 The Office of Scientific Research, Hebei Normal University of Science and Technology, Qinhuangdao, Hebei, China; 2 Engineering Research Center of Chestnut Industry Technology, Ministry of Education, Hebei Normal University of Science and Technology, Qinhuangdao, Hebei, China; 3 CHN Energy Qinghai Electric Power Co., Ltd., Xining, Qinghai, China; 4 PowerChina Northwest Engineering Corporation Limited, Xi’an, Shaanxi, China; 5 College of Electrical Engineering and New Energy, China Three Gorges University, Yichang, Hubei, China; 6 Research Institute of Forestry, Chinese Academy of Forestry Sciences, Beijing, China

**Keywords:** *Castanea mollissima*, GATA, phenotype, RNA-Seq, RT-qPCR, salt stress

## Abstract

The *GATA* gene family comprises important transcription factors (TFs) that affect plant ontogeny and abiotic/biotic stress tolerance. *Castanea mollissima* is a significant economic and food-producing tree species in the Northern Hemisphere, known for its strong wind resistance and soil stabilization capabilities. Nevertheless, existing studies have not explored the characteristics and functions of the *GATA* family in *C. mollissima*. The study identified 21 *CmGATA* genes from the N11–1 reference genome assembly of *C. mollissima*, which were dispersed among 12 chromosomes. Phylogenetic analysis helped to categorize these genes into four distinct subfamilies, with genes belonging to the same subfamily exhibiting considerable similarities in terms of structural characteristics and conserved motif composition. Collinear analysis further revealed that the major driver of *CmGATA* gene family expansion was segmental duplication, followed by evolutionary pressure predominantly shaped by purifying selection. Phenotypic indicators, such as antioxidant enzyme activity, exhibited significant alterations, and transcriptomic analysis revealed increasingly more differentially expressed genes (DEGs) as salt stress treatment continued. Notably, transcriptome analysis demonstrated the expression patterns of *CmGATA* genes under salt stress, and combined with their correlation with phenotype indicators and weighted gene co-expression network analysis (WGCNA), effectively benefiting the exploration of the function of *CmGATA* genes in *C. mollissima* salt stress response. Additionally, validation experiments utilizing reverse transcription-quantitative polymerase chain reaction (RT-qPCR) demonstrated gene expression trends consistent with those obtained from RNA sequencing (RNA-seq) analyses. This study theoretically assists in comprehending the evolution and function of the *CmGATA* genes under salt stress, offering potential applications in environmental conservation and saline–alkali land improvement. Enhancing salt tolerance in *C. mollissima* could support its cultivation in a wider range of regions, combining ecological benefits of soil and water conservation with economic value.

## Introduction

Transcription factors (TFs) are DNA-binding proteins that regulate plant growth, development, and stress responses by activating or repressing target gene transcription (Riechmann et al., 2000). Several TF families like WRKY, MYB, NAC, and bHLH are systematically characterized in plants due to their critical regulatory roles (Li et al., 2021; Liu et al., 2023; Wang et al., 2023; Yu et al., 2023). GATA TFs are broadly distributed among plants, exhibiting a type IV zinc finger domain (CX_2_CX_17-20_CX_2_C) and an adjacent basic region (Bose et al., 2025). In angiosperms, *GATA* genes are classified into four subgroups, three containing 18 residues and one containing 20 residues in the zinc finger loop (Schwechheimer et al., 2022a). These zinc finger proteins excel in recognizing specific *cis*-elements to dynamically modulate gene expression (Wu et al., 2025).

Researchers have found members of this family in numerous plant species, including *Glycine max* (64) (Zhang et al., 2015), *Triticum aestivum* (79) (Du et al., 2022), *Cucumis sativus* (26) (Zhang et al., 2021), *Capsicum annuum* (28) (Yu et al., 2021), *Cucumis melo* (24) (Zheng et al., 2024), *Citrus sinensis* (24) (Fan et al., 2024), and *Populus trichocarpa* (39) (An et al., 2020). According to studies, GATA TFs essentially impact plant ontogeny and abiotic/biotic stress tolerance. Specifically, GNC, a GATA TF in *A. thaliana*, dynamically regulates histone modifications and DNA methylation by directly binding to target genes and recruiting chromatin-remodeling complexes, thereby integrating environmental cues with developmental processes and controlling flowering time (Yang et al., 2022). Under high-nitrogen conditions, *CsGATA17* acts as a transcriptional activator that directly upregulates *CsAlaDC* expression, promoting theanine biosynthesis in *C. sinensis* leaves (Gao et al., 2025). *OsGATA12* suppresses chlorophyll degradation genes through its zinc finger domain, delays leaf senescence, and regulates tillering, thereby improving *O. sativa* yield by enhancing photosynthetic efficiency and resource distribution (Lu et al., 2017). Moreover, *OsGATA7* modulates grain morphology and plant architecture by integrating brassinosteroid (BR) signaling (Zhang et al., 2018). Two GATA members in *T. aestivum*, *TaGATA62* and *TaGATA73*, interact with light-signaling proteins through zinc finger domains to regulate antioxidant enzyme activity, maintaining redox homeostasis and enhancing drought and salt tolerance in *Saccharomyces cerevisiae* and *A. thaliana* (Du et al., 2022). *GmGATA44* overexpression in *A. thaliana* rescues the reduced chlorophyll phenotype of the *AtGATA21* mutant (Zhang et al., 2015). In *C. sinensis*, overexpression of *CsGATA12* markedly decreases resistance to citrus bacterial canker (Fan et al., 2024).

*Castanea mollissima*, a deciduous species of the Fagaceae family, is a valuable food and cash crop in the Northern Hemisphere attributed to its nutrient-rich kernels (Huang et al., 2023). However, its productivity and industrial development are severely constrained by various environmental stresses, including temperature, drought, salinity, and gall wasp (*Dryocosmus kuriphilus*) infestation. *C. mollissima* is primarily cultivated in mountainous areas and wastelands, where soil salinization/alkalization can severely affect its growth and productivity. Recent genome-wide studies in *C. mollissima* have begun to elucidate the molecular mechanisms underlying abiotic stress responses, including the glutaredoxin (GRX) gene family (Liu et al., 2026) and the aldehyde dehydrogenase (ALDH) superfamily (Wang et al., 2026), both of which have been implicated in salinity stress responses. However, the GATA TF family, which plays key roles in salt stress responses in other plant species, remains unexplored in *C. mollissima*. Salt stress represents an important abiotic constraint adversely impacting plant growth and developmental processes (Luo and Christie, 2002). Given the critical involvement of the GATA TF family in mediating plant responses and adaptations to saline conditions, systematically characterizing these genes in *C. mollissima* is essential to uncover their potential impact on salt stress tolerance (Bose et al., 2025). Therefore, the current study provided a detailed exploration of *CmGATA* genes, encompassing protein physicochemical characteristics, evolutionary relationships, genomic structures, *ci*s-regulatory elements, TF regulatory networks, and patterns of gene duplication. To elucidate molecular mechanisms underlying the salt stress response in *C. mollissima*, plants underwent salt treatment, after which transcriptomic profiles and physiological parameters, including antioxidant enzyme activities, were measured. Interestingly, combined with weighted gene co-expression network analysis (WGCNA), there is a clear linkage between the expression of some *CmGATA* genes and physiological changes under salt stress. All these theoretically assist in elucidating the functional significance of *GATA* genes in plant stress adaptation.

## Materials and methods

### Identification, structural, and phylogenetic analyses

The genome sequence data, annotated genome structure files (GFF3 format), and protein sequences for *C. mollissima* were acquired from the Castanea Genome Database (http://castaneadb.net/). The N11–1 reference genome assembly was used for all bioinformatic analyses in this study. To identify GATA proteins in the *C. mollissima* proteome, BLAST searches were performed using 30 *A. thaliana* GATA protein sequences from TAIR as queries, with an *E*-value cutoff of 1e−5. Candidate sequences with sequence identity ≥30% were retained. The Pfam database provided the conserved GATA domain (PF00320), which was used alongside the HMMER 3.0 software to screen candidate CmGATA proteins from BLAST alignment outcomes. The NCBI-Conserved Domain Database (CDD) and Simple Modular Architecture Research Tool (SMART) databases were applied to the examination of putative CmGATA proteins to ascertain the presence of conserved GATA domains. Analysis of the physicochemical characteristics, conserved motifs, and predicted subcellular localizations of CmGATA proteins was conducted with Expasy, MEME, and WoLF PSORT tools, respectively. GATA proteins derived from *C. mollissima*, *A. thaliana*, and *O. sativa* underwent multiple sequence alignment using MEGA 7.0, and a phylogenetic tree was subsequently generated by employing the maximum likelihood (ML) method with 1,000 bootstrap replicates to evaluate nodal support. Protein secondary structures were predicted by SOPMA, while SWISS-MODEL was employed for three-dimensional structural modeling of CmGATA proteins. To evaluate potential assembly-specific biases, we extended our GATA gene identification to all seven available *C. mollissima* genome assemblies: N11-1, HBY2, HSZ7, Sun, Vanuxem, Yanhong (YH), and Yanshanzaofeng (YSZS). For each assembly, *GATA* genes were identified following the same pipeline described above, including BLASTP searches, domain validation using Pfam (PF00320) and NCBI-CDD, and phylogenetic analysis using MEGA 7.0 with 1,000 bootstrap replicates. Gene structures and chromosomal distributions were visualized using TBtools.

### Collinear analysis of *CmGATAs*

Information regarding the chromosomal positioning of *CmGATA* genes was acquired from the GFF3 annotation file, alongside the visualization utilizing the TBtools software (Chen et al., 2020). Moreover, MCScanX software was used for identifying collinear relationships within the *C. mollissima* genome and between *C. mollissima* and *A. thaliana*, *Quercus robur*, *Solanum lycopersicum*, *Vitis vinifera*, *Zea mays*, *Oryza sativa*, and *Pyrus bretschneideri* (Wang et al., 2012). The genome datasets for these additional species were obtained from the Phytozome database. These seven species were selected to cover major angiosperm lineages while also considering their phylogenetic relationships with *C. mollissima*. Specifically, *Q. robur* (Fagaceae) was included as a closely related species within the same family, while *P. bretschneideri* (Rosaceae), belonging to the same order (Rosales) but a different family, represents a more distantly related woody dicot. In addition, *A. thaliana* and *S. lycopersicum* represent distant dicots, *V. vinifera* represents a basal eudicot, and *O. sativa* and *Z. mays* represent monocots. This combination provides a broad evolutionary framework—from closely related to distantly related lineages—for understanding GATA gene family evolution. Duplication events involving *CmGATA* genes were categorized by employing the “duplicate_gene_classifier” module of MCScanX. Whole-genome duplication events and segmental duplications were further distinguished based on analyses of collinear dot-plots, calculations of synonymous substitution rates (*K*_s_) between gene pairs, and evaluations of collinear block complementarity (Yu et al., 2025a).

### Promoter analysis and functional annotation

The promoter sequences (2,000 bp upstream of transcription start sites) of *CmGATA* genes were extracted from the *C. mollissima* genome annotation file via TBtools, and PlantCARE was employed for the identification of potential *cis*-acting elements (Lescot et al., 2002). Additionally, the Plant Transcriptional Regulatory Map (PTRM) tool served for predicting TFs potentially interacting with *CmGATA* gene promoters. Cytoscape software was employed for relevant visualization (Kohl et al., 2011). Functional annotation and enrichment analyses relied on eggNOG-mapper and TBtools, respectively (Cantalapiedra et al., 2021).

### Plant materials and phenotype determination

The *C. mollissima* cultivar “Yanshanzaofeng”, grown for 60 days prior to treatment, served as the experimental material during the summer of 2025. Three plants underwent a salt stress treatment of 200 mmol/L NaCl solution, while another three received regular irrigation with water, serving as controls; treatments were administered every 10 days. The NaCl concentration was selected based on previous studies on salt stress responses in *C. mollissima* and other woody plants (Huaxin et al., 2008; Li et al., 2024). Physiological parameters, including soil electrical conductivity (EC) and leaf pigmentation characteristics, were assessed on days 0, 14, and 26 following treatment initiation. Leaf tissues from both control and salt-stressed plants were subjected to liquid nitrogen freezing, and then preserved at −80 °C pending RNA sequencing and physiological analyses. Soil EC was evaluated using an LY610 Yi-Xin soil detector (Guangzhou Zhonghong Instrument Co., Ltd., China). Leaf color characteristics were assessed with a Minolta Chroma Meter CR-200 (Konica Minolta Holdings, Inc., Japan). The CIELAB color parameters L*, a*, and b* were recorded, where L* indicates lightness (0 = black, 100 = white), a* indicates the red–green axis (positive = red, negative = green), and b* indicates the yellow–blue axis (positive = yellow, negative = blue). Malondialdehyde (MDA) content and superoxide dismutase (SOD) and peroxidase (POD) activities were measured using commercial assay kits (Keming, Suzhou, China) according to the manufacturer’s instructions. Proline content was measured via the sulfosalicylic acid method (Elasad et al., 2020).

### RNA-seq analysis

Total RNA isolation from leaf tissues was conducted with the CTAB-PBIOZOL reagent protocol, followed by ethanol precipitation (Kiss et al., 2024). RNA quality assessment and quantification were performed utilizing an Agilent 2100 Bioanalyzer and a NanoDrop spectrophotometer (Thermo Fisher Scientific, MA, USA). The cDNA libraries were subsequently constructed employing the NEBNext^®^ Ultra™ RNA Library Prep Kit (Illumina, San Diego, CA, USA). We deposited the generated RNA sequencing data into the NCBI repository (Bioproject PRJNA1363574). HISAT2 software (version 2.0.5) was utilized to map clean sequencing reads to the N-11 reference genome of *C. mollissima*, as previously outlined (Kim et al., 2019; Tian et al., 2025; Yu et al., 2025a, Yu et al., 2025b). Gene expression quantification was carried out using FeatureCounts, and expression levels were normalized and presented as FPKM (Fragments Per Kilobase of transcript per Million mapped reads) values (Liao et al., 2014). DEGs between groups were identified via DESeq2 (version 1.22.1) with the following criteria: |log_2_ fold change| ≥ 1 and FDR < 0.05, following Benjamini–Hochberg correction for multiple hypothesis testing.

### WGCNA and RT-qPCR validation

WGCNA (the MetWare Cloud platform) adopted a 0.25 threshold for dendrogram module merging and a 0.6 correlation coefficient cutoff for heatmap visualization. MetWare Biotechnology (Wuhan, China) synthesized gene-specific primers designed via Primer Premier 5 software, as detailed in **Supplementary Table S1** (Singh et al., 1998). Reverse transcription-quantitative polymerase chain reaction (RT-qPCR) experiments were performed using a Bio-Rad CFX Manager Real-Time PCR system (Bio-Rad Laboratories, USA). Each 10-μL reaction contained 5 μL of 2× SG Green qPCR Mix, 0.5 μL each of forward and reverse primers (10 μM), 1 μL of cDNA, and 3 μL of nuclease-free water. The thermal protocol was as follows: 95 °C for 3 min; 40 cycles of 95°C for 10 s, 60 °C for 20 s, and 72 °C for 10 s. Melting curve analysis was performed for all reactions to confirm specific single-product amplification (**Supplementary Figure S1**). No amplification was detected in non-template controls (NTCs). *Actin* was selected as the reference gene because it has been systematically validated as one of the most stable internal controls across various tissues of *C. mollissima* (Chen et al., 2019), and its suitability for salt stress studies in this species has been confirmed in recent transcriptomic and RT-qPCR analyses (Liu et al., 2026; Wang et al., 2026). Its stable amplification across our samples was confirmed by the low CT variation observed in our preliminary RT-qPCR runs, and the comparative CT method (2^−ΔΔCt^) (Livak and Schmittgen, 2001) was applied to calculating the relative gene expression.

## Results

### Molecular features and evolutionary relationships of *CmGATA* genes

The study identified 21 *GATA* genes in the *C. mollissima* genome considering conserved domain similarity (**Supplementary Table S2**). CmGATA proteins contain 132 (CmGATA18) to 544 residues (CmGATA26), with molecular weights of 14.50 kDa (CmGATA16) to 60.38 kDa (CmGATA26). Physicochemical analyses predicted all CmGATA proteins to be unstable hydrophobic proteins, with an average hydrophobicity index below zero and an instability index above 40. The aliphatic index was 50.03 (CmGATA2b) to 74.08 (CmGATA25b), and theoretical isoelectric points were 4.74 (CmGATA28c) to 10.26 (CmGATA23). Subcellular localization analysis predicted all CmGATA proteins to localize to the nucleus. Secondary structure prediction ascertained random coils as the predominant structural component, ranging from 62.12% (CmGATA18) to 89.85% (CmGATA8a), averaging 82.09%. The α-helix content ranged from 6.47% (CmGATA28b) to 33.53% (CmGATA23), averaging 14.11%, while extended strands ranged from 1.79% (CmGATA12a) to 6.06% (CmGATA18) (**Supplementary Table S3**). Predicted three-dimensional structures indicated high similarity and conservation among CmGATA proteins (**Supplementary Figure S2**). A phylogenetic tree constructed from GATA protein sequences of *C. mollissima*, *A. thaliana*, and *O. sativa* divided CmGATA proteins into four subfamilies (**Figure 1**; **Supplementary Table S4**). The 21 CmGATA members were unevenly distributed among these subfamilies, with subfamily I containing the highest number (11), followed by subfamily III (5), subfamily II (4), and subfamily IV containing only one member.

**Figure 1:**
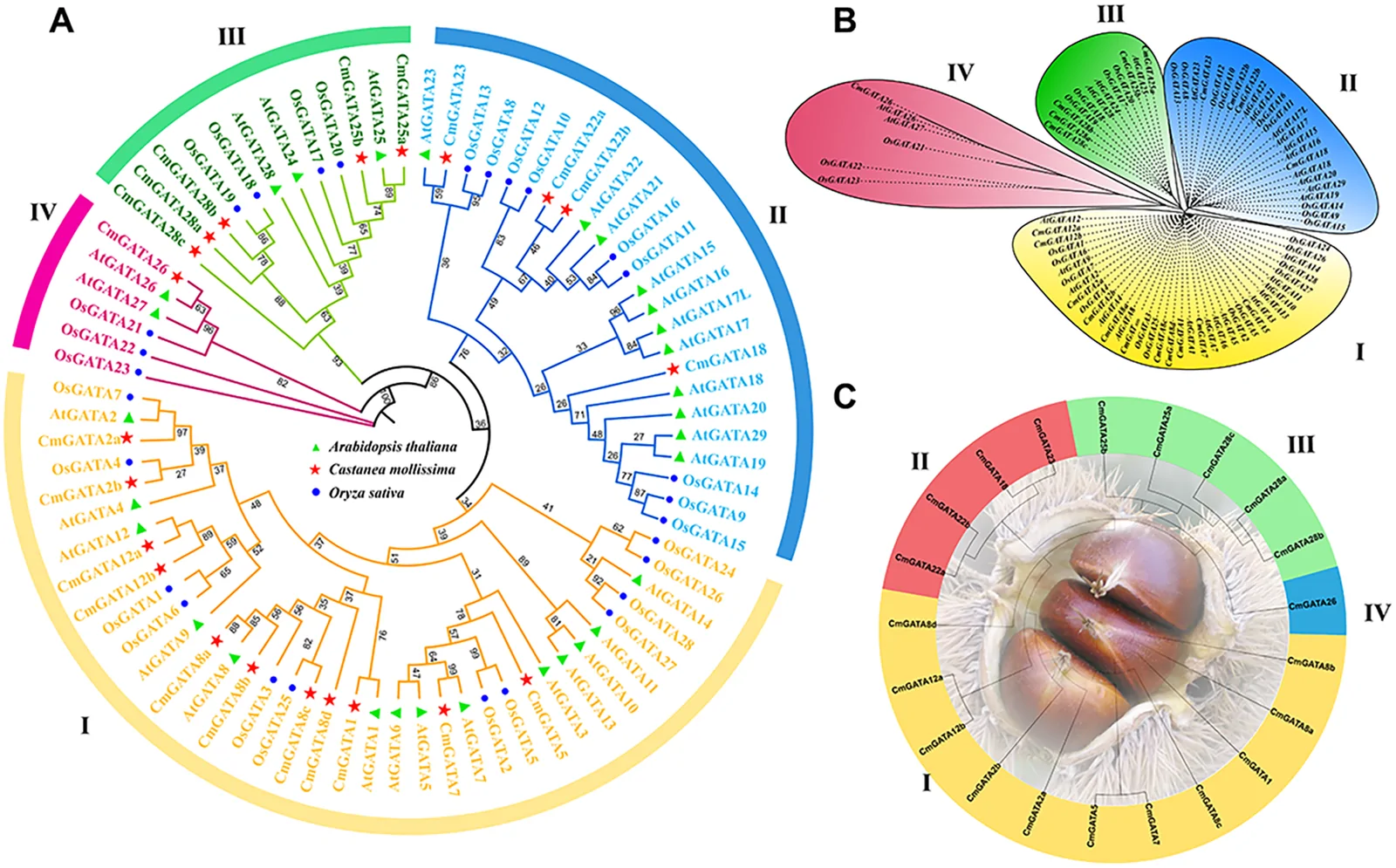
Phylogenetic relationships of *GATA* genes among *C. mollissima*, *A. thaliana*, and *O. sativa*. **(A)** Rooted tree with bootstrap support values (1,000 replicates) indicated at each node. **(B)** Unrooted tree of the GATA family. **(C)** Rooted topology specifically for *C. mollissima*.

### Characterization of *CmGATAs*

The molecular architecture of *CmGATA* genes—including exon–intron organization, domain conservation, and motif composition—is illustrated in **Figure 2** and **Supplementary Figure S3**. All CmGATA protein sequences contained a single conserved GATA domain, demonstrating strong conservation. Interestingly, phylogenetic analysis revealed additional CCT and TIFY domains in subfamily III members and a DEUBAD_NFRKB domain in subfamily IV members. *CmGATA* genes contained between 1 and 10 introns (**Figure 2**; **Supplementary Table S2**), with subfamily III having the most (average 7.8 introns), subfamilies I and II fewer (average 1.7 introns), and subfamily IV only one member (7 introns). Notably, all CmGATA proteins shared motifs 1 and 5, corresponding to the GATA domain (**Figure 2**). Moreover, subfamilies I, II, and IV harbor 18 residues in the zinc-loop, whereas subfamily III has 20. Certain amino acid sites, like “LCNACG”, were highly conserved, conforming to findings in other plant GATA proteins (Reyes et al., 2004; Ma et al., 2025). Overall, members of each subfamily shared similar features in both motif arrangement and gene architecture, which agrees with the phylogenetic grouping.

**Figure 2:**
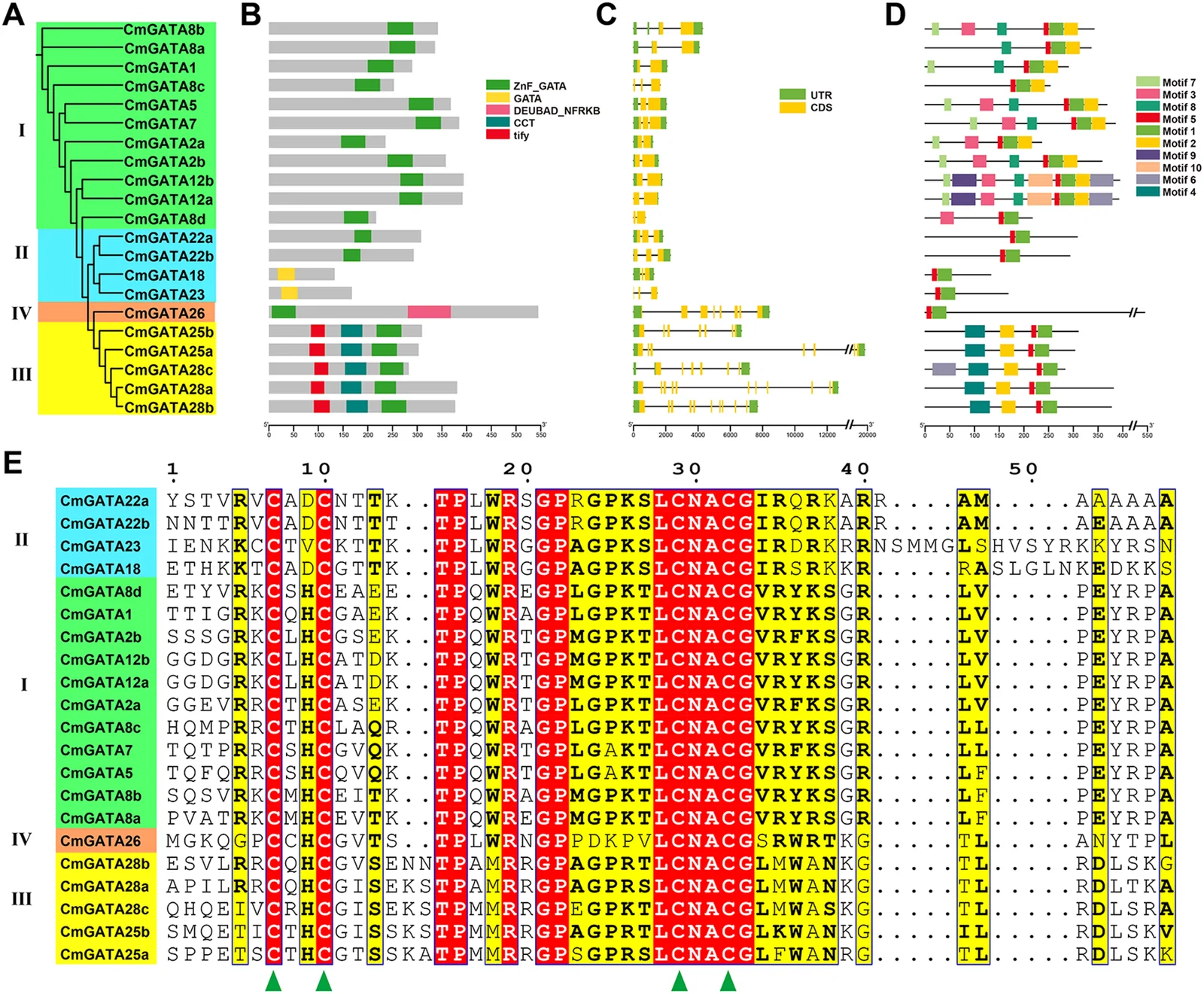
Sequence and structural features of CmGATA proteins and genes. **(A)** Phylogenetic reconstruction. **(B)** Conserved domain architecture. **(C)** Exon–intron organization. **(D)** Motif composition. **(E)** Multiple alignment of the CmGATA domain.

### Duplication analysis of *CmGATAs*

The 21 *CmGATA* genes demonstrated an uneven distribution across the 12 chromosomes (**Figure 3A**). Chromosomes 8, 11, and 12 each contained three *CmGATA* genes, chromosomes 1, 2, and 10 each contained two *CmGATA* genes, while the remaining chromosomes each possessed one *CmGATA* gene. A gene family comprises genes with similar structures and functions derived from a common ancestral gene through duplication events (Domazet-Lošo et al., 2024). Gene duplication significantly drives gene family expansion and functional diversification (Innan and Kondrashov, 2010; Shan et al., 2025). We classified duplication types of *CmGATA* genes using the “duplicate_gene_classifier” function in MCScanX software (**Figure 3B**). By integrating orthologous alignment analysis with *K*_s_ value calculations, WGD events and segmental duplications were clearly distinguished (Yu et al., 2022). Eight *CmGATA* members originated from segmental duplication, seven from dispersed duplication, two from WGD, two from tandem duplication, and two from proximal duplication (**Figure 3C**). Furthermore, *K*_a_/*K*_s_ values for *CmGATA* homologous gene pairs were <1, indicating effective purifying selection (**Supplementary Table S5**).

**Figure 3:**
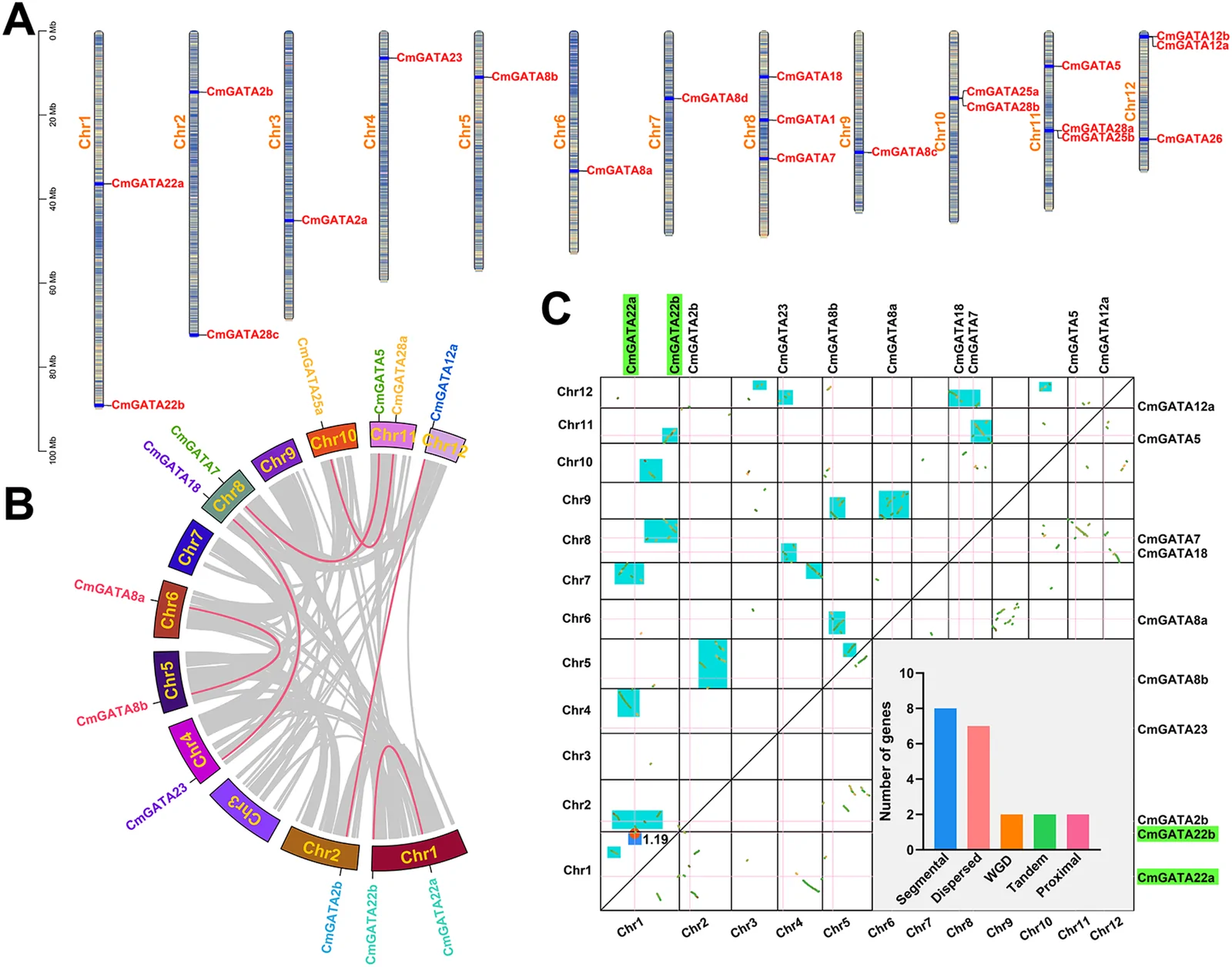
Duplication-type analysis of *CmGATA* genes. **(A)** Chromosomal positions. **(B)** Intragenomic collinear within the *C. mollissima* genome. **(C)** Collinear dot-plot highlighting WGD-derived blocks, with block lengths and median *K*_s_ values indicated. Inset bar chart shows duplication-type frequencies.

### Cross-assembly conservation of the GATA gene family

To assess potential assembly-specific biases, we extended our *GATA* gene identification to seven available *C. mollissima* genome assemblies: N11-1, HBY2, HSZ7, Sun, Vanuxem, YH, and YSZS. The number of *GATA* genes identified ranged from 21 to 25 across assemblies (**Supplementary Figure S4**), with N11–1 containing 21 members, HBY2 containing 23, HSZ7 containing 22, Sun containing 25, Vanuxem containing 21, YH containing 21, and YSZS containing 25. Phylogenetic analysis of GATA proteins from all seven assemblies (**Supplementary Figure S5**) demonstrated that the core *GATA* gene complement is largely conserved across different assemblies. Chromosomal distribution and gene structure analysis (**Supplementary Figures S6**, **S7**) further confirmed the overall conservation of *GATA* genes across assemblies, with minor variations attributable to differences in assembly quality, annotation completeness, and potentially genuine intra-specific copy number variations among cultivars. These results confirm that the 21 *CmGATA* genes identified in the N11–1 assembly represent the core GATA family size for *C. mollissima*.

### Genomic collinear and gene retention patterns of the GATA family

Collinear analysis elucidates the arrangement and distribution of homologous genes within or between species, thereby elucidating their origin and evolutionary relationships (Wang et al., 2012). The present study conducted collinear analysis between *C. mollissima* and seven representative species to clarify the evolutionary history of the GATA gene family (**Figure 4**). The analysis identified massive homologous gene pairs containing *CmGATA* across these genomes: *Q. robur* (12), *P. bretschneideri* (27), *V. vinifera* (22), *A. thaliana* (21), *S. lycopersicum* (32), *O. sativa* (6), and *Z. mays* (2). Correspondingly, these homologous gene pairs involved 11, 14, 15, 12, 13, 5, and 2 *CmGATA* genes, respectively. Notably, certain *CmGATA* genes formed multiple gene pairs with homologous genes from other plants. For instance, *CmGATA7* formed homologous pairs with four genes each in *P. bretschneideri* and *S. lycopersicum*, while *CmGATA18* paired with four genes each in *A. thaliana* and *S. lycopersicum* (**Supplementary Tables S6–S12**). These results suggested that *CmGATA7* and *CmGATA18* retained more gene copies in other species. According to the gene balance hypothesis, genes related to macromolecular complexes or signaling pathways are typically retained due to dosage sensitivity (Birchler and Veitia, 2007). Thus, the retention of multiple copies of *CmGATA7* and *CmGATA18* indicates their potentially significant roles in complex molecular or signaling pathways. Additionally, *CmGATA*s formed more homologous gene pairs with dicotyledons compared with monocotyledons, suggesting that *CmGATA* genes may have undergone additional duplication events following monocot–dicot divergence (**Figure 4B**). Furthermore, statistical analysis of collinear blocks containing *CmGATA* genes indicated more numerous and longer collinear blocks between *C. mollissima* and dicotyledons compared with monocotyledons (**Figure 4C**; **Supplementary Tables S13–S19**). However, statistical analyses did not clearly correlate with evolutionary relationships among these plants. Previous studies on other gene families in *C. mollissima* reported similar results; hence, these discrepancies may arise from specific evolutionary events (e.g., WGD) in varying plant species (Tian et al., 2025; Yu et al., 2025b).

**Figure 4:**
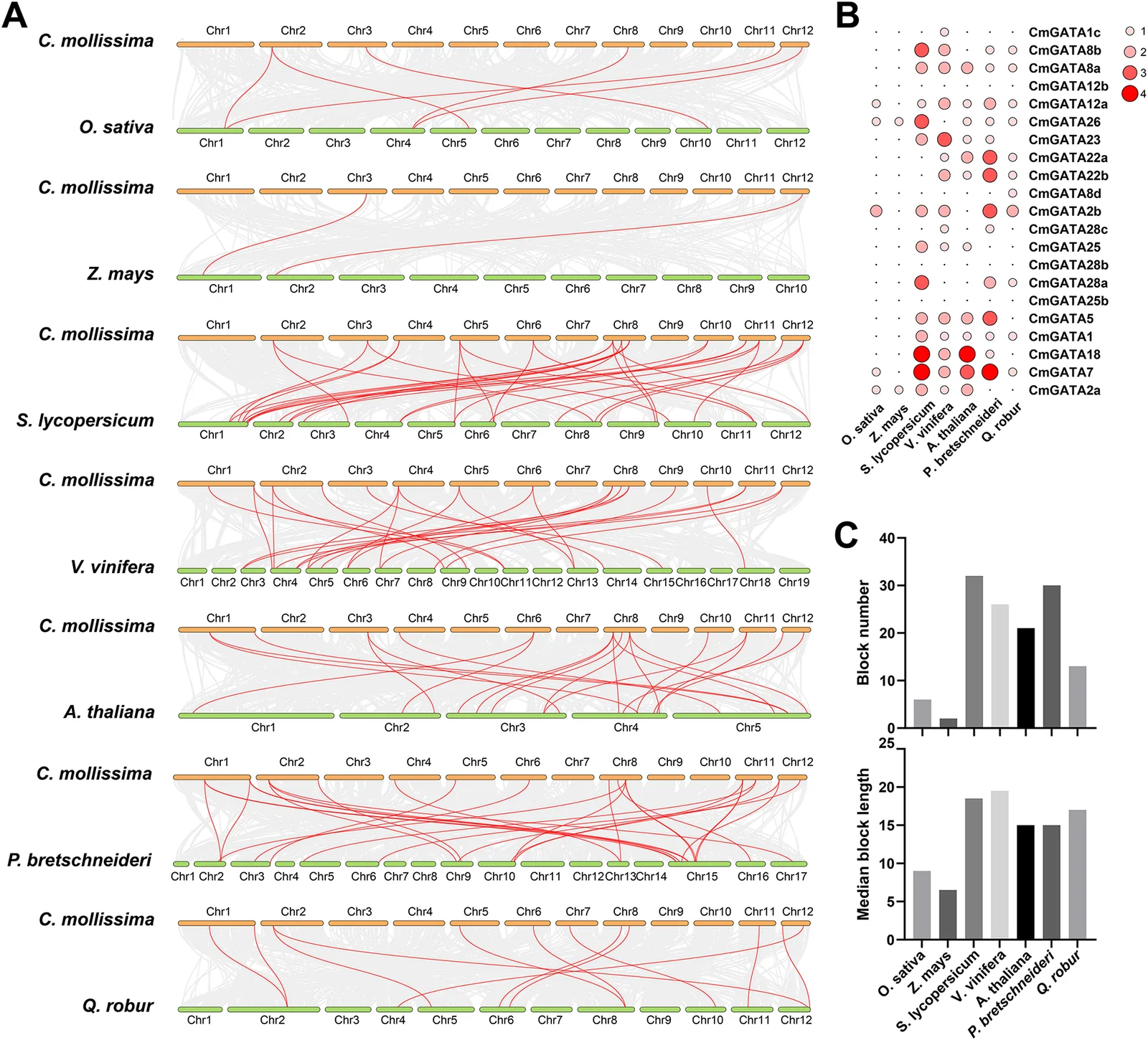
Cross-species collinear of *CmGATAs* with seven representative plants. **(A)** Dual-species collinear plots (gray: background collinear blocks; red: GATA–GATA orthologs). **(B)** Orthologous gene-pair counts. **(C)** Number of collinear blocks and their median lengths.

### Promoter analysis and functional annotation

Five hundred twenty-nine *cis*-acting elements identified in the promoters of 21 *CmGATA* genes fell into four groups: hormone-responsive, environmental stress-related, development-related, and light-responsive (**Figure 5**; **Supplementary Table S20**). Notably, certain *cis*-elements of abscisic acid-responsive (ABRE), methyl jasmonate-responsive (CGTCA), and light-responsive (Box4 and G-box) elements, were present in nearly all *CmGATA* promoters. Additional *cis*-elements associated with environmental stress were identified, such as drought-responsive (MBS) and anaerobic inducibility (GC-motif) elements. Furthermore, 612 TFs predicted to regulate *CmGATA* promoters belonged to 43 TF families (**Figure 6A**; **Supplementary Table S21**). Among these families, ERF had the highest number (80 members), followed by MYB (67), NAC (59), WRKY (43), and bZIP (35), suggesting that these TF families might play key roles in regulating *CmGATA* genes (**Figure 6B**). Additionally, as revealed by Kyoto Encyclopedia of Genes and Genomes (KEGG) enrichment analysis, *CmGATA* genes were primarily enriched in “TFs” and “protein families: genetic information processing”. Gene Ontology (GO) enrichment analysis highlighted “regulation of transcription by RNA polymerase II”, “positive regulation of transcription”, and “response to abiotic stimulus” (**Figures 6C, D**). These findings align with established mechanisms of GATA-regulated gene expression (Reyes et al., 2004; Bose et al., 2025; Wu et al., 2025). Collectively, functional enrichment, *cis*-element identification, TF regulatory network analysis, and subsequent transcriptomic and WGCNAs comprehensively elucidate the potential functions of *CmGATA* genes.

**Figure 5:**
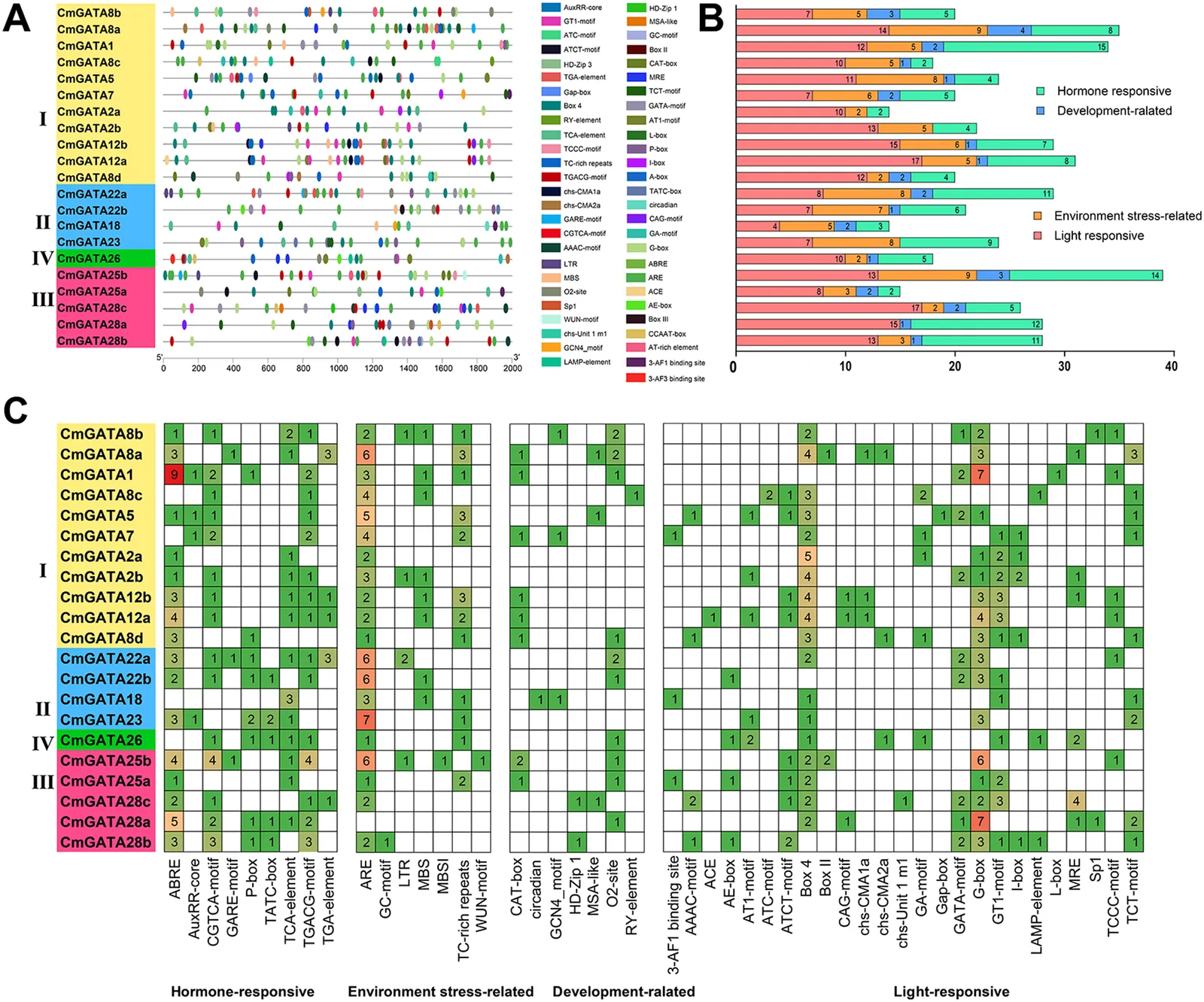
*Cis*-regulatory elements in *CmGATA* promoter regions. **(A)** Element distribution map, where colored symbols mark individual elements and their relative positions. **(B)** Proportional composition of elements (same color = similar/identical functions). **(C)** Absolute counts of distinct element types.

**Figure 6:**
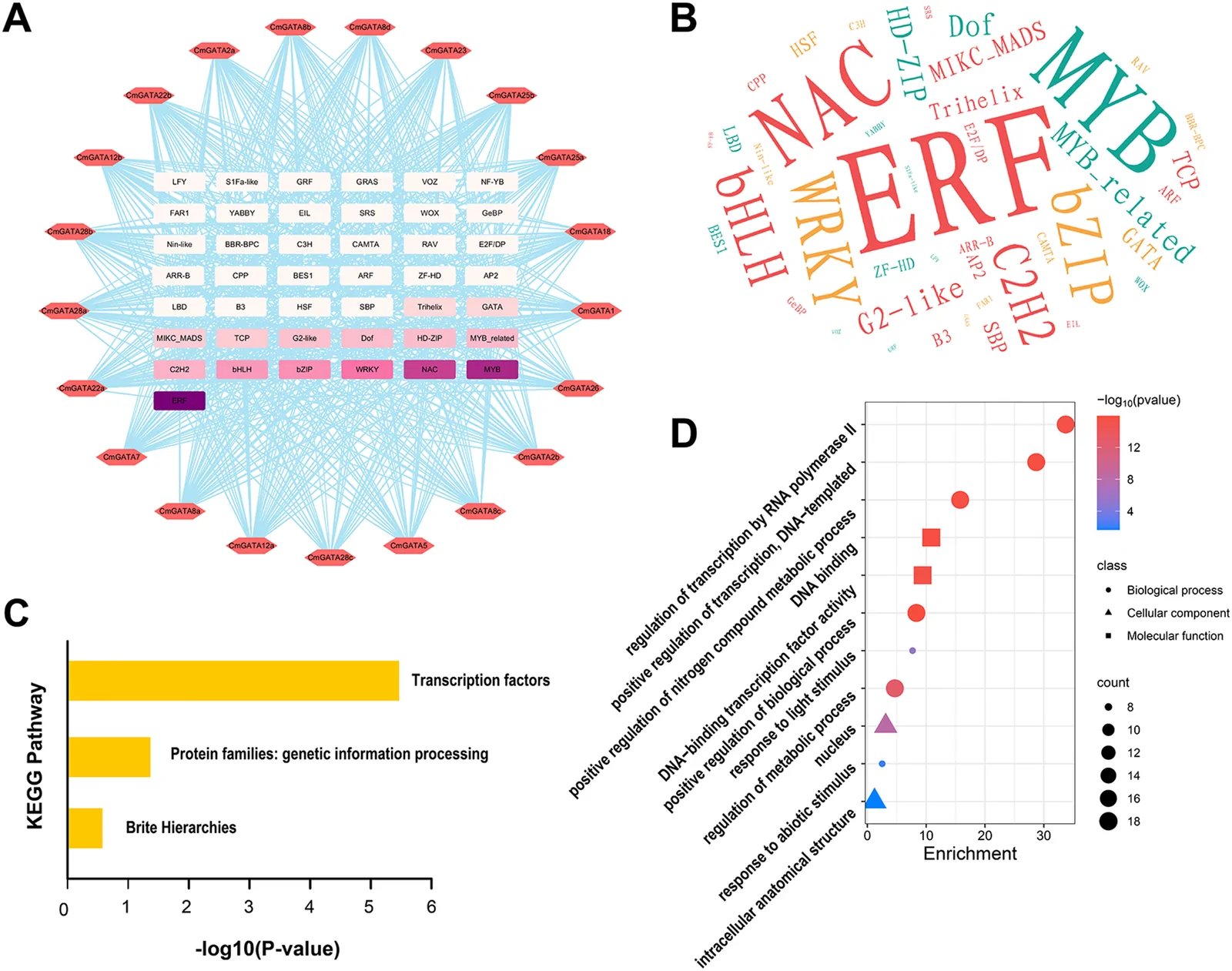
Regulatory networks and functional annotation of *CmGATA* genes. **(A)** Predicted transcription factor (TF) interaction network. **(B)** Word cloud of putative TFs, with font size proportional to TF occurrence frequency. **(C, D)** KEGG pathway and GO term enrichment results.

### Morphological and physiological characteristics of *C. mollissima* under salt stress

Salt stress was applied by irrigating plants with a 200 mmol/L NaCl solution (**Figure 7A**). Changes in soil EC between control and salt-treated groups are presented in **Figure 7B**. After 14 and 26 days of treatment, soil EC significantly increased from 0.09 to 2.11 and 3.70 mS/cm, respectively. The EC of control soil, irrigated with normal water, remained stable (0.16–0.22 mS/cm). These results indicated effective salt stress application.

**Figure 7:**
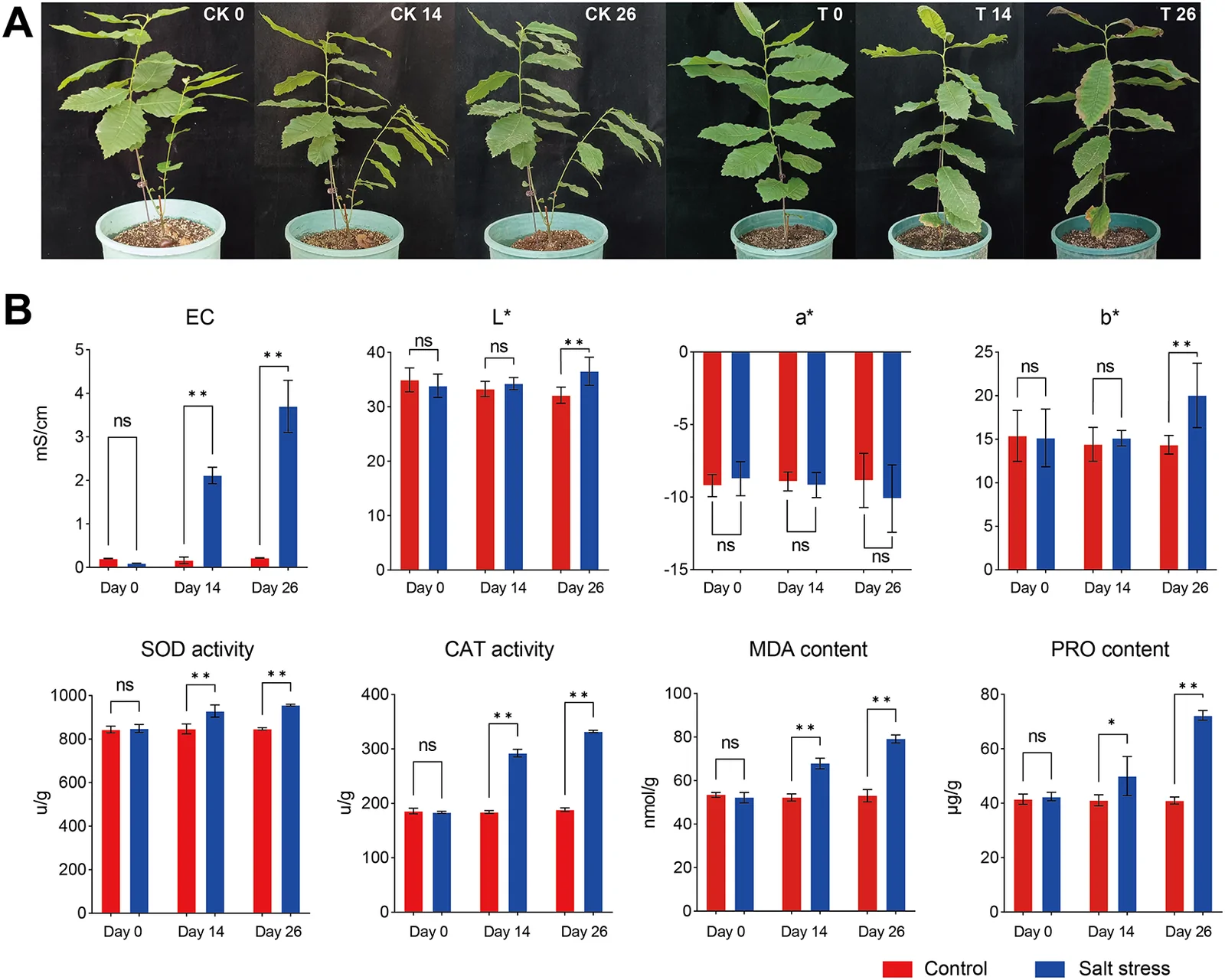
Variation of the morphology and physiological index of *C. mollissima* under salt stress treatments. **(A)** The *C. mollissima* plants. **(B)** Soil electrical conductivity and change in leaf color (L*, a*, and b*), SOD and CAT activity, MDA content, and proline content in *C. mollissima* leaves under salt stress treatment. One-way ANOVA served for analyzing the statistical significance (*p < 0.05, **p < 0.01; ns, not significant).

Relative to the control, salt stress remarkably affected the morphology of *C. mollissima* plants, primarily causing leaf wilting at the plant tops (**Figure 7A**). To quantitatively evaluate leaf color changes, leaf color parameters were measured. Parameter a* (red–green axis) did not significantly change under salt stress; however, parameters L* (lightness) and b* (yellow–blue axis) significantly increased compared with the control after 26 days (**Figure 7B**). Physiological responses, including SOD and CAT activities, MDA content, and proline levels, were further evaluated. Plants produce ROS under salt stress, adversely affecting growth (Mittler et al., 2022). Superoxide radicals (O_2_^−^) are dismutated into hydrogen peroxide and oxygen under SOD catalysis (Wang et al., 2018). Since hydrogen peroxide can damage cells, it can be decomposed into water and oxygen under CAT catalysis, protecting cells from oxidative damage. Following 14 and 26 days of salt stress, SOD activity significantly rose from 848.97 to 928.94 and 956.18 U/g, respectively, and CAT activity remarkably increased from 183.66 to 292.39 and 332.23 U/g, respectively. Proline, an essential osmolyte, scavenges ROS to mitigate oxidative stress (OS) (Hayat et al., 2012). Under salt stress, proline content significantly increased from 42.26 to 50.00 μg/g (day 14) and 72.28 μg/g (day 26). MDA is commonly used to indicate OS and membrane damage (Guan et al., 2023; Zuo et al., 2025). Salt stress significantly elevated MDA content from 52.23 nmol/g (control) to 67.86 nmol/g (day 14), and further to 79.13 nmol/g (day 26), suggesting prolonged oxidative damage in *C. mollissima* plants.

### RNA sequencing, DEG identification, and WGCNA

To elucidate the transcriptional regulatory mechanisms underlying salt tolerance in *C. mollissima*, leaf samples were collected from salt-stressed plants at 14 days (D14T) and 26 days (D26T) post-treatment, alongside corresponding control groups (D14CK and D26CK). Twelve cDNA libraries were generated, with three biological replicates for each experimental condition. Sequencing yielded a total of 98.36 Gb of high-quality clean reads, exhibiting Q20 and Q30 values above 99% and 97%, respectively. The GC content remained consistent, ranging from 43.19% to 43.56% (**Supplementary Table S22**). According to PCA, the transcriptomic profiles of different treatment groups were clearly separated (**Figure 8A**). Furthermore, the read alignment rate to the reference genome varied between 91.46% and 91.78% (**Supplementary Table S23**). Collectively, the obtained transcriptomic data exhibit high reliability, integrity, and quality.

**Figure 8:**
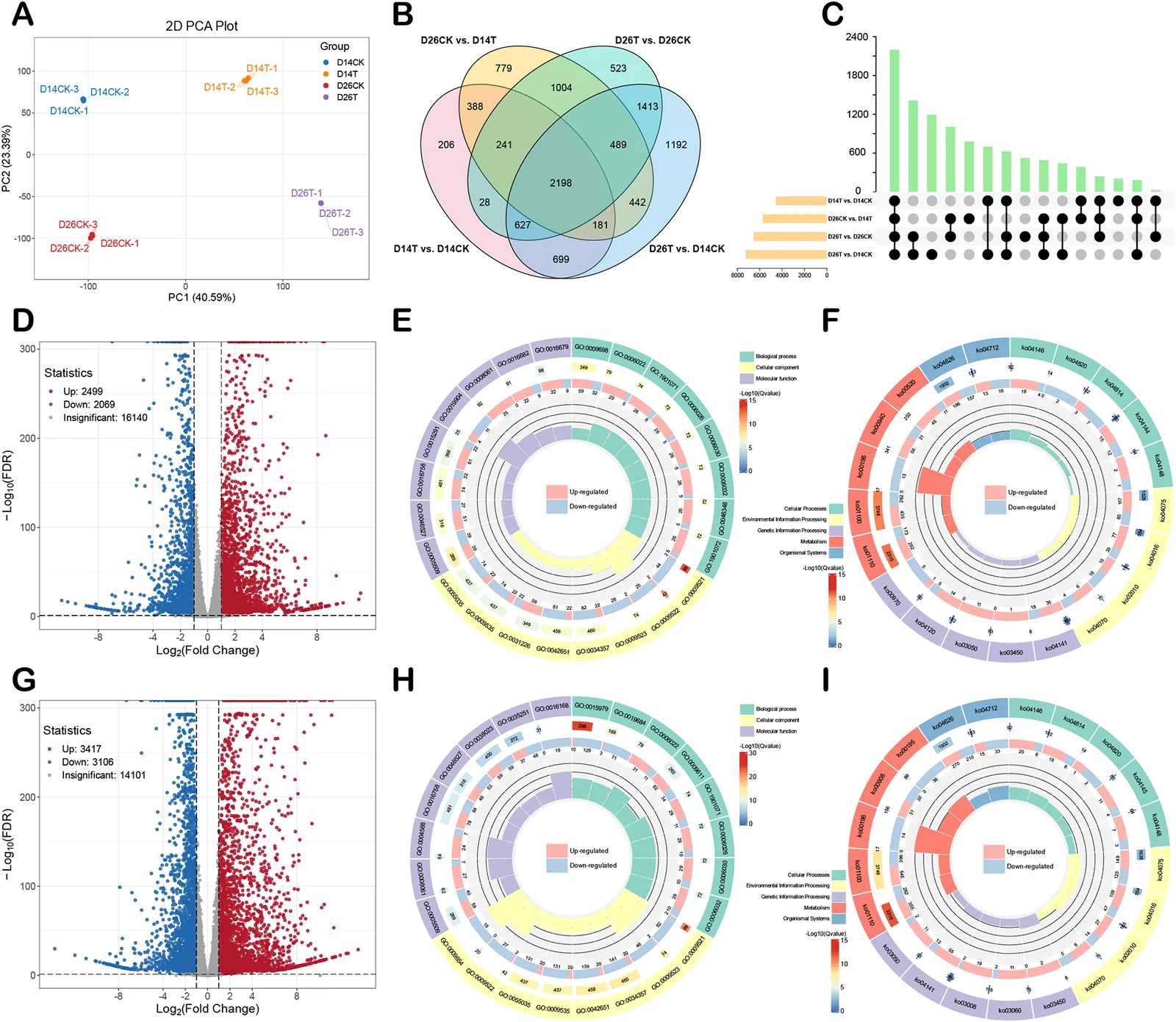
Overview of transcriptomic profiling. **(A)** PCA clustering of all samples. **(B, C)** Distribution of differentially expressed genes (DEGs) across four treatment comparisons. **(D)** DEGs identified in D14T vs. D14CK, with corresponding GO and KEGG enrichment shown in **(E, F)**. **(G)** DEGs for D26T vs. D26CK, with functional enrichment presented in **(H, I)**.

Comparative analysis identified 10,410 DEGs among the four treatment groups (**Figures 8B, C**). Specifically, comparisons (D14T vs. D14CK and D26T vs. D26CK) identified 2,499 and 3,526 upregulated genes, respectively, along with 2,069 and 3,715 downregulated genes (**Figures 8D, G**). Hence, the number of DEGs is positively associated with salt stress duration, suggesting that gene activation intensifies with increasing stress exposure. GO and KEGG analyses revealed similar enrichment in functional categories and pathways associated with hormone signaling and environmental stress responses (**Figures 8E, F, H, I**).

WGCNA was conducted using the transcriptomic and phenotypic data to identify vital modules and core genes driving plants’ salt stress responses (**Figure 9A**). Thirteen modules displayed distinct correlations with various phenotypic indicators under salt stress (**Figure 9B**). For example, the yellow module showed a negative correlation with SOD activity (*r* = −0.95, *p* = 2.3 × 10^−6^), CAT activity (*r* = −0.99, *p* = 7.7 × 10^−10^), and MDA content (*r* = −0.98, *p* = 2.4 × 10^−8^), while the turquoise module positively correlated with SOD activity (*r* = 0.93, *p* = 1.2 × 10^−5^), CAT activity (*r* = 0.97, *p* = 1.8 × 10^−7^), MDA content (*r* = 0.98, *p* = 2.4 × 10^−8^), and proline content (*r* = 0.94, *p* = 5.5 × 10^−6^). Genes within the yellow and turquoise modules participate differently in salt stress responses. According to functional enrichment analyses, the yellow module associates with photosynthesis, pigment biosynthesis, and energy metabolism, while the turquoise module associates predominantly with protein processing, vesicle-mediated transport, and endomembrane organization, suggesting distinct biological roles during salt stress responses (**Figures 9C–F**). Notably, CmGATA5, CmGATA8a, CmGATA32, CmGATA25b, and CmGATA28c localized within the yellow and turquoise modules, implying their strong involvement in *C. mollissima* salt tolerance.

**Figure 9:**
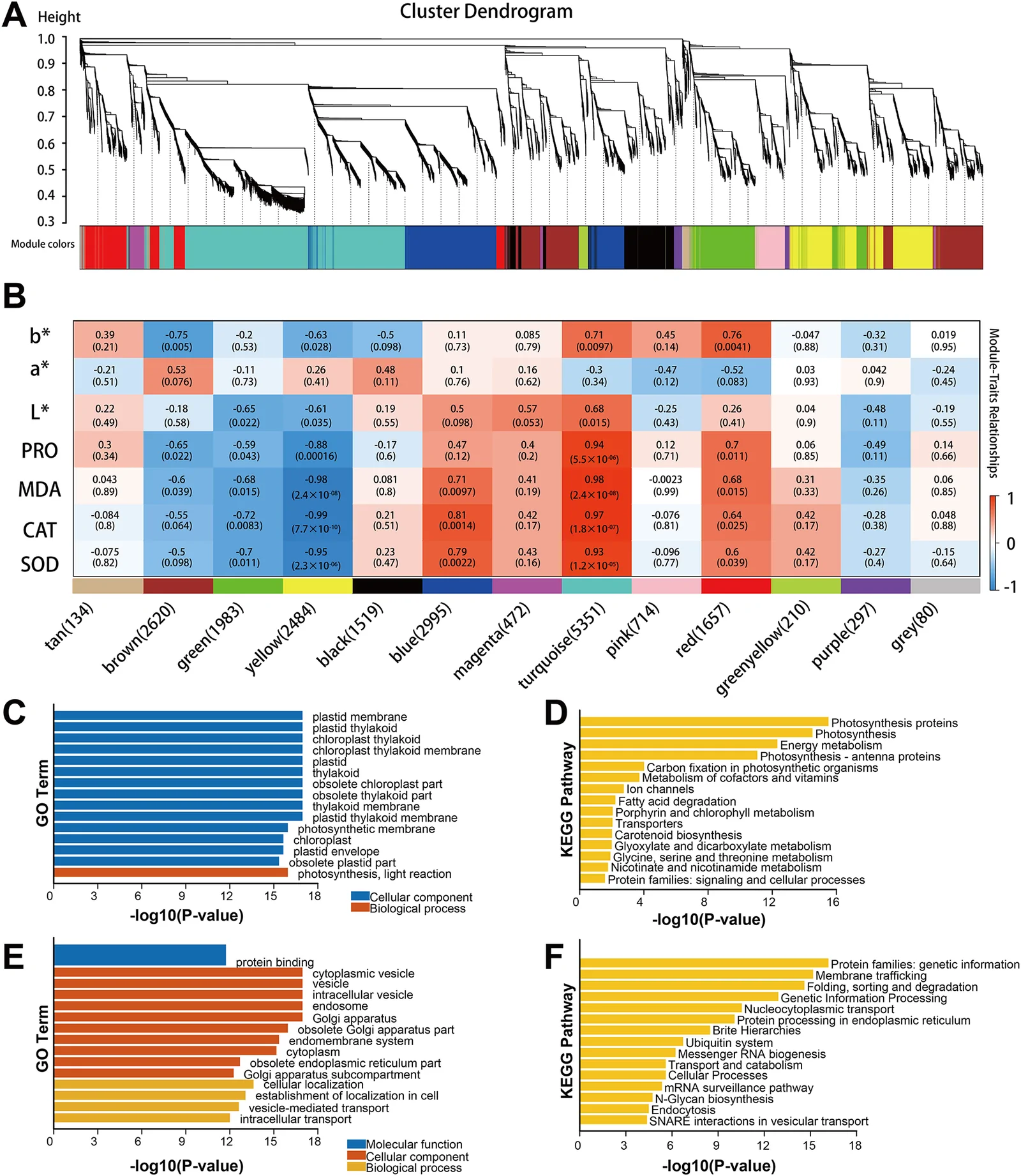
Identification and functional profiling of WGCNA modules. **(A)** Gene dendrogram used for module definition. **(B)** Correlative relationships between each module and developmental traits. GO and KEGG enrichment analyses of **(C, D)** the yellow modules and **(E, F)** the turquoise modules.

### Correlation between *CmGATAs* and phenotypic indicators, and RT-qPCR validation

Melting curve analysis revealed a single sharp peak for each primer pair across all tested samples, confirming specific single-product amplification (**Supplementary Figure S1**). Several *CmGATA* genes showed significant differential expression between control and salt-stressed conditions (**Figures 10A, B**). Certain genes, including *CmGATA5*, *CmGATA28c*, *CmGATA25a*, and *CmGATA28b*, exhibited marked induction under salt stress. For instance, the FPKM value of *CmGATA5* increased from 76.77 to 98.34 after 14 days, and further rose from 56.23 to 112.72 after 26 days of salt stress. Conversely, some genes, including *CmGATA18*, *CmGATA28c*, and *CmGATA5*, exhibited significantly reduced expression levels under salt stress. To explore the relationship between these expression patterns and physiological responses, correlations between *CmGATA* gene expression levels and key phenotypic indicators were analyzed (**Figure 10C**). *CmGATA28c* exhibited significant positive correlations with MDA and proline contents, while *CmGATA25b* and *CmGATA12a* negatively correlated with SOD, CAT activities, and MDA content. For validating RNA sequencing (RNA-seq) accuracy, RT-qPCR analysis was conducted on four *CmGATA* genes showing significant expression changes under salt stress (**Figure 11**), with results displaying high consistency with the transcriptomic data, ascertaining the reliability of *CmGATA* gene regulation patterns in *C. mollissima* under salt stress.

**Figure 10:**
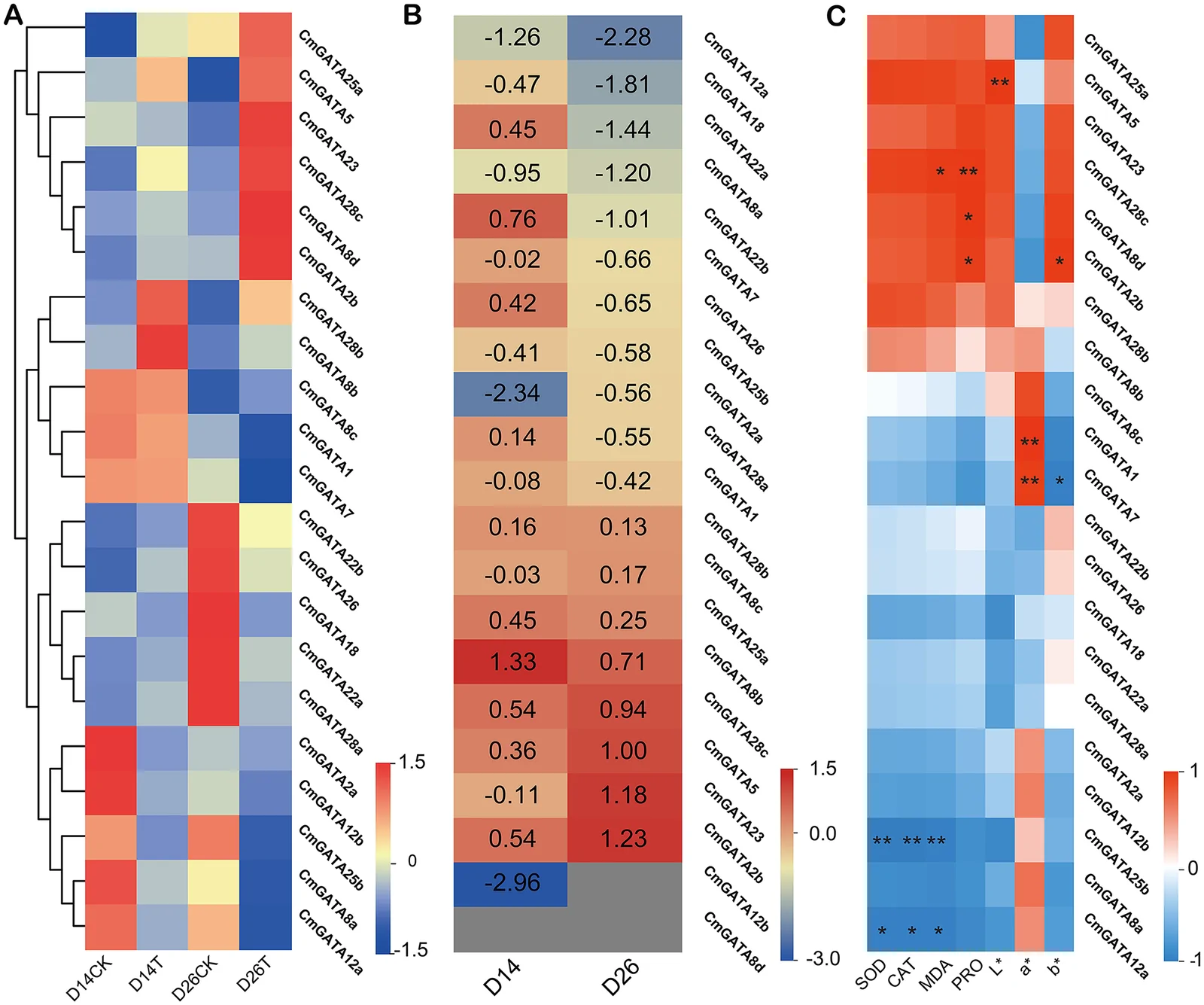
Expression profiles of *CmGATA* genes and their associations with physiological traits under salt stress. **(A)** Heatmap of FPKM expression levels of 21 *CmGATA* genes across all samples. **(B)** Heatmap of log_2_ fold change values of *CmGATA* genes under salt stress at 14 and 26 days (treatment vs. control), with numerical values displayed in each cell. Red indicates upregulation, blue indicates downregulation, white indicates no change, and gray indicates that log_2_FC could not be calculated due to zero FPKM values. **(C)** Correlations between *CmGATA* gene expression and phenotypic parameters under salt treatments. The color scale represents Pearson correlation coefficients; *p < 0.05, **p < 0.01, ***p < 0.001.

**Figure 11:**
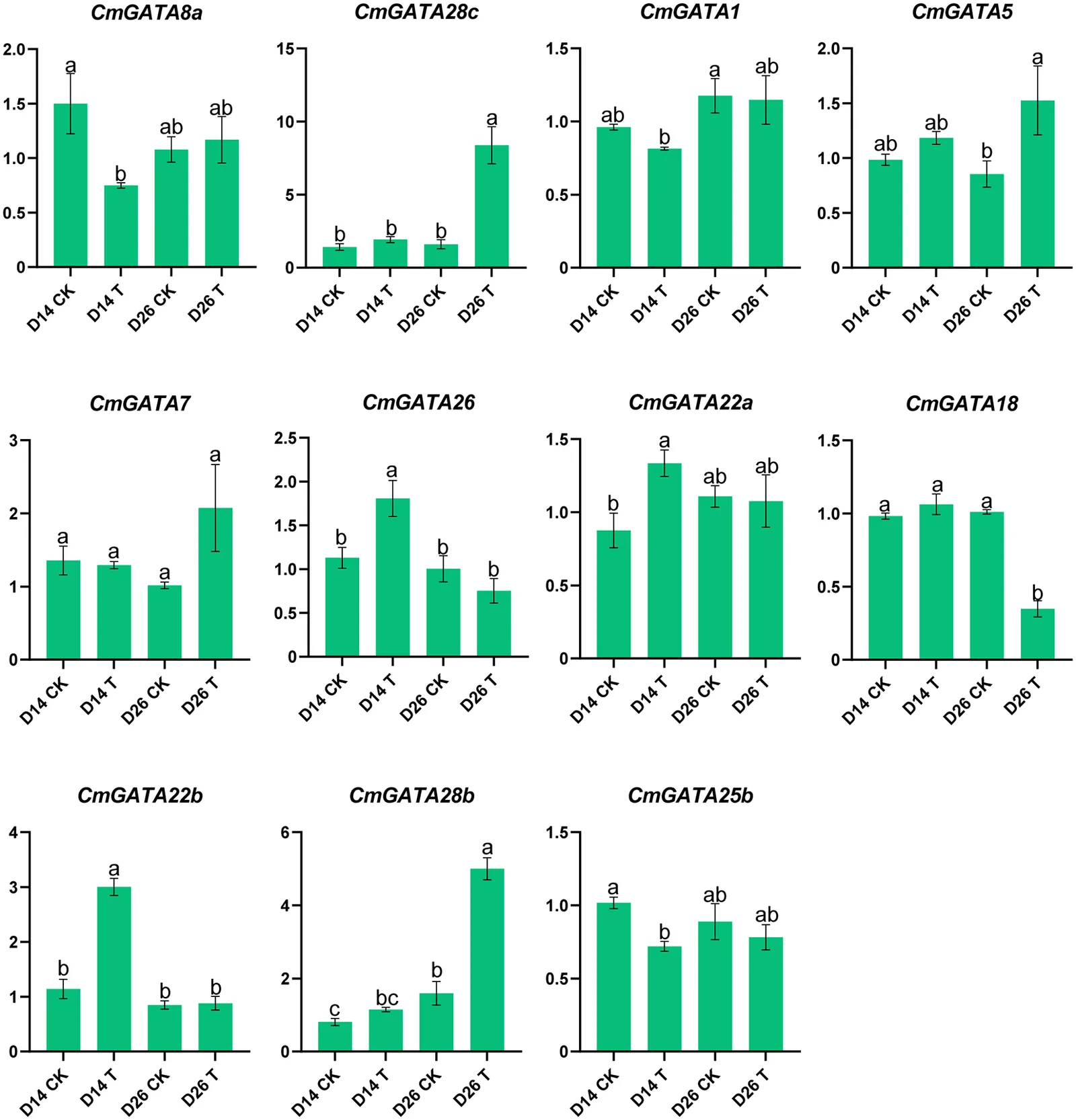
RT-qPCR validation of selected *CmGATAs*. Lowercase letters indicate significant differences among RT-qPCR data, respectively.

## Discussion

Plant TFs participate in various physiological and biochemical processes during plant growth and development (Viswanath et al., 2023). Despite the investigation into the structure and functions of the GATA family in many plant species, their distribution and functional characteristics in *C. mollissima* remain poorly understood (Zhang et al., 2021; Gao et al., 2025). This study is the first one comprehensively analyzing the *C. mollissima* GATA gene family, revealing potential roles in salt stress responses and theoretically benefiting the future functional research.

### Molecular characteristics of *CmGATAs*

The study identified 21 *CmGATA* genes from the N11–1 reference genome assembly of *C. mollissima* genome. Bioinformatics analyses predicted that these genes encode hydrophilic, acidic nuclear-localized proteins, most of which are unstable (**Supplementary Table S2**). Generally, hydrophilic proteins diffuse more easily within cells, while acidic proteins are more likely to interact with positively charged DNA-binding domains (Bienert and Chaumont, 2014; Hossain et al., 2023). Additionally, unstable proteins enable rapid responses to environmental stress and can be rapidly degraded by specific enzymes, preventing excessive activation of downstream genes (Clausen et al., 2019). CmGATA proteins exhibit similar physicochemical properties to GATA proteins in other plants of *Citrus sinensis* (Fan et al., 2024) and *C. melo* (Zheng et al., 2024). All CmGATA proteins contain the conserved GATA zinc-finger domain, classified into four typical angiosperm subfamilies (**Figure 2**) (Schwechheimer et al., 2022a). As observed in other plant species, *CmGATA* genes within the same subfamily show highly similar gene structure, conserved domains, and motif arrangements (Peng et al., 2021; Yao et al., 2023). Notably, *CmGATA* members in subfamily III contain both CCT and TIFY domains. The CCT domain integrates light signals and circadian rhythms through DNA binding and protein interactions, thereby regulating developmental timing (Griffiths et al., 2003). The TIFY domain coordinates the balance between stress response and development through jasmonic acid signaling (Chung and Howe, 2009; Thireault et al., 2015; Huang et al., 2017). Collectively, subfamily III *CmGATA*s might regulate development and stress responses in *C. mollissima*. Additionally, *CmGATA* members in subfamilies I and II possess fewer and shorter introns (**Figure 2**). Shorter introns typically correlate with higher gene expression levels, reducing transcriptional and splicing costs (Castillo-Davis et al., 2002; Heyn et al., 2015). Furthermore, compared to genes rich in introns, intron-poor genes frequently respond to drought and salt stress in *A. thaliana* (Liu et al., 2021). Transcriptomic analyses indicated that certain members of subfamilies I and II, like *CmGATA5* and *CmGATA8d*, exhibited higher expression under salt stress, further suggesting their involvement in stress responses (**Figure 10A**). Additionally, various hormone and stress-responsive *cis*-elements (ABRE, P-box, LTR, MBS, TGA-element, and WUN-motif) were identified in *CmGATA* promoter regions (**Figure 5**). Interaction network analyses further indicated potential regulatory relationships between *CmGATA* genes and TFs such as MYB, GRAS, bHLH, and NAC (**Figure 6**). These findings demonstrate that *CmGATA* genes crucially dominate *C. mollissima* growth, development, and environmental stress adaptation through complex transcriptional regulatory networks.

### Evolution and expansion of *CmGATAs*

The GATA gene family originated from single-cell eukaryotes. GATA TFs (such as *GAT1* and *GLN3* in *S. cerevisiae*) already existed in lower eukaryotes, participating primarily in fundamental biological processes like nitrogen metabolism and metal ion homeostasis (Kulkarni et al., 2006). This study identified 21 *CmGATA* genes in *C. mollissima*, fewer than in species such as *G. max* (64) (Zhang et al., 2015) and *P. trichocarpa* (39) (An et al., 2020), but comparable to *O. sativa* (28) (Reyes et al., 2004) and *C. sativus* (26) (Zhang et al., 2021). Indeed, there are much more *GATA* genes in plants versus fungi and animals, primarily due to species-specific gene duplication events (Schwechheimer et al., 2022a). The expansion of *GATA* genes and significant copy-number differences across plant genomes are attributed mainly to various duplication events (Schwechheimer et al., 2022a; Bose et al., 2025; Ma et al., 2025). Collinear analyses between *C. mollissima* and five dicotyledonous and two monocotyledonous plants provided insights into the evolutionary trajectory of angiosperm GATA genes (**Figure 4**). The collinear relationships between *C. mollissima* and dicots were notably stronger, especially regarding the number and length of collinear blocks containing homologous *CmGATA* gene pairs (**Figure 4C**; **Supplementary Tables S13–S19**). However, an intriguing finding emerged when comparing collinear relationships among the five dicots and *C. mollissima*: metrics such as the number and length of collinear blocks did not significantly correlate with evolutionary distances among species. That conforms to studies of other gene families in *C. mollissima*, including the ABC and DnaJ families (Yu et al., 2025a, Yu et al., 2025b). This phenomenon likely arises from genomic reshuffling in different plant species under distinct ecological pressures, leading to gene loss or retention of beneficial mutations. Such reshuffling affects the detection of collinear regions and their gene compositions (Barakat et al., 2009; Yang et al., 2021; Wang et al., 2024). Additionally, varying genome assembly quality among species may influence intergenomic collinearity (Yu et al., 2025a). Within the *C. mollissima* genome, 38.10% and 33.33% of *CmGATA* genes underwent segmental and dispersed duplication, respectively, highlighting their importance in expanding the *CmGATA* gene family (**Figure 3C**). The essential roles of segmental and dispersed duplication in gene-family expansion in plants are well documented (Cannon et al., 2004; Chen et al., 2023; Sun et al., 2023). Segmental duplications preserve complete gene structures and regulatory sequences, facilitating maintenance of original gene functions (Vollger et al., 2022). Dispersed duplications relocate gene copies within genomes, releasing them from original regulatory constraints and enabling the evolution of new functions, particularly common in stress-responsive gene families (Chen et al., 2023; Yu et al., 2025a, Yu et al., 2025b).

### *CmGATA* gene responses to salt stress

Salt stress is a primary abiotic factor dominating plant growth and development. Plants strengthen salt tolerance by activating stress-related genes regulated by TFs (Zhang et al., 2023; Abdulla et al., 2024). Through transcriptomic analyses and RT-qPCR validation, multiple *CmGATA* genes displayed significant expression changes under salt stress, suggesting their involvement in regulating salt responses in *C. mollissima* (**Figures 8**, **10**). For example, *CmGATA5*, *CmGATA28c*, *CmGATA25a*, and *CmGATA28b* exhibited markedly elevated expression under salt stress, particularly at 26 days post-treatment. Specifically, the expression of *CmGATA5* increased significantly from 56.23 to 112.72 FPKM, indicating a robust induction. Conversely, genes such as *CmGATA18* and *CmGATA28c* showed decreased expression under salt stress, suggesting possible functional differentiation among *CmGATA* family members.

According to correlation analyses, expression levels of certain *CmGATA* genes significantly correlated with SOD, CAT, and proline (**Figure 10**). For instance, *CmGATA28c* exhibited significant positive correlations with MDA and proline content, while *CmGATA25b* and *CmGATA12a* negatively correlated with SOD, CAT activities, and MDA content. These results suggest that *CmGATA* genes may regulate antioxidant systems and osmotic balance under salt stress conditions. Additionally, WGCNA grouped several *CmGATA* genes into modules (yellow and turquoise) strongly correlated with salt-stress phenotypes, further supporting their central roles in salt tolerance (**Figure 9**).

The *cis*-acting elements within promoter regions help to reveal the gene regulatory mechanisms. Promoters of *CmGATA* genes contain abundant elements of ABRE, LTR, and MBS, related to abiotic stress responses (salt, drought, and low temperature) and hormonal signals (ABA and JA) (**Figure 5**). This indicates precise regulation of *CmGATA* gene expression by complex upstream signaling networks. Furthermore, based on predicted TF regulatory networks, members of key stress-related TF families (ERF, MYB, NAC, and WRKY) potentially regulate *CmGATA* expression directly (**Figure 6**). Therefore, we hypothesize that salt stress activates these TFs, which then regulate specific *CmGATA* genes. Subsequently, CmGATA proteins interact with additional factors to jointly regulate downstream effectors involved in OS defense and osmotic adjustment, ultimately forming a transcriptional regulatory cascade that enhances *C. mollissima* salt stress adaptation.

Only a single salt concentration and two late time points (14 and 26 days) were examined in this study, and that our *cis*-element analysis was descriptive in nature. Future investigations incorporating a salinity gradient, early time points, and statistical enrichment analysis of *cis*-elements would provide a more comprehensive understanding of *CmGATA* gene regulation under salt stress. Furthermore, the RNA-seq dataset generated in this study provides an opportunity for identifying single nucleotide polymorphisms (SNPs) and InDels within CmGATA transcripts, which could serve as molecular markers for salt tolerance breeding in *C. mollissima*.

In summary, after systematically identifying the GATA TF family in *C. mollissima*, this study comprehensively analyzed its molecular characteristics, evolutionary expansion, and regulation under salt stress. The findings reveal fundamental features of *CmGATA* genes and provide substantial evidence supporting their critical roles in salt stress responses. Specifically, *CmGATA5* and *CmGATA28c* represent priority candidates for functional validation via overexpression or gene editing approaches. These results establish a foundation for future genetic improvement of salt tolerance in *C. mollissima*.

## Data Availability

The datasets presented in this study can be found in online repositories. The names of the repository/repositories and accession number(s) can be found in the article/**Supplementary Material**.

## Supplementary material

Supplementary Figure 1 Melting curves confirming single-product amplification for all gene-specific primer pairs and the *Actin* reference gene.

Supplementary Figure 2 The predicted 3D structure of CmGATA proteins.

Supplementary Figure 3 Distribution of conserved motifs of CmGATA proteins.

Supplementary Figure 4 Summary of GATA gene family member numbers identified across seven *C. mollissima* genome assemblies.

Supplementary Figure 5 Phylogenetic analysis of GATA proteins identified across seven *C. mollissima* genome assemblies, showing the evolutionary relationships and conservation of GATA members among different assemblies.

Supplementary Figure 6 Chromosomal distribution of *GATA* genes across seven *C. mollissima* genome assemblies.

Supplementary Figure 7 Gene structure of *GATA* genes across seven *C. mollissima* genome assemblies.
